# The Matrix Stiffness Coordinates the Cell Proliferation and PD-L1 Expression via YAP in Lung Adenocarcinoma

**DOI:** 10.3390/cancers16030598

**Published:** 2024-01-31

**Authors:** Yeonhee Park, Dahye Lee, Jeong Eun Lee, Hee Sun Park, Sung Soo Jung, Dongil Park, Da Hyun Kang, Song-I Lee, Seong-Dae Woo, Chaeuk Chung

**Affiliations:** 1 Division of Pulmonary and Critical Care Medicine, Department of Internal Medicine, Daejeon St. Mary’s Hospital, College of Medicine, The Catholic University of Korea, Seoul 34943, Republic of Korea; yhpark@catholic.ac.kr; 2 Division of Pulmonology and Critical Care Medicine, Department of Internal Medicine, College of Medicine, Chungnam National University, Daejeon 34134, Republic of Korea; ziczi02@naver.com (D.L.); jelee0210@cnu.ac.kr (J.E.L.); sparkylove@cnuh.co.kr (H.S.P.); sungsoojung09@cnu.ac.kr (S.S.J.); rahms@cnuh.co.kr (D.P.); ibelieveu113@cnuh.co.kr (D.H.K.); songi_cnuh@cnuh.co.kr (S.-I.L.); nextera@cnuh.co.kr (S.-D.W.)

**Keywords:** lung adenocarcinoma, matrix stiffness, PD-L1, YAP

## Abstract

**Simple Summary:**

Tumor development is often accompanied by abnormal extracellular matrix (ECM) remodeling and increased stiffness. A stiff ECM promotes mechanotransduction, and the predominant transcription factors implicated in this phenomenon are YAP/TAZ, β-catenin, and NF-κB. We sought to investigate whether YAP acts as a critical mediator between matrix stiffness and PD-L1 in lung adenocarcinoma. We confirmed that YAP, PD-L1, and Ki-67, a marker of cell proliferation, increased as the matrix stiffness increased in vitro. Through YAP knockdown and overexpression in a stiff-matrix environment, we discovered that YAP expression levels have an impact on the expression of PD-L1 and Ki-67. This study highlights the critical role of YAP in linking the ECM to PD-L1.

**Abstract:**

The extracellular matrix (ECM) exerts physiological activity, facilitates cell-to-cell communication, promotes cell proliferation and metastasis, and provides mechanical support for tumor cells. The development of solid tumors is often associated with increased stiffness. A stiff ECM promotes mechanotransduction, and the predominant transcription factors implicated in this phenomenon are YAP/TAZ, β-catenin, and NF-κB. In this study, we aimed to investigate whether YAP is a critical mediator linking matrix stiffness and PD-L1 in lung adenocarcinoma. We confirmed that YAP, PD-L1, and Ki-67, a marker of cell proliferation, increase as the matrix stiffness increases in vitro using the lung adenocarcinoma cell lines PC9 and HCC827 cells. The knockdown of YAP decreased the expression of PD-L1 and Ki-67, and conversely, the overexpression of YAP increased the expression of PD-L1 and K-67 in a stiff-matrix environment (20.0 kPa). Additionally, lung cancer cells were cultured in a 3D environment, which provides a more physiologically relevant setting, and compared to the results obtained from 2D culture. Similar to the findings in 2D culture, it was confirmed that YAP influenced the expression of PD-L1 and K-67 in the 3D culture experiment. Our results suggest that matrix stiffness controls PD-L1 expression via YAP activation, ultimately contributing to cell proliferation.

## 1. Introduction

The formation and development of tumor cells are closely linked to the microenvironment in which the tumor grows. The tumor microenvironment (TME) comprises several major components, including cancer-associated fibroblasts (CAFs), immune cells, vasculature, tumor-associated endothelial cells, and the extracellular matrix (ECM) [1]. The ECM plays an important role in cell-to-cell communication, tumor cell proliferation, and metastasis, as well as in the mechanical support of tumor cells [2]. Aberrations in the ECM promote tumor angiogenesis and inflammation, resulting in a tumorigenic microenvironment [3]. The development of solid tumors is often associated with the abnormal cross-linking, remodeling, and increased stiffness of the ECM [4]. The stiffness of solid lung cancers (20–30 kPa) is significantly higher than that of normal lung parenchyma (0.5–5 kPa) [5]. A stiff ECM triggers mechanotransduction, which involves the conversion of mechanical cues, such as the stiffness of the matrix, into biochemical signals in the cells. Transcription factors critical to these processes include Yes-associated protein (YAP)/transcriptional coactivator with PDZ-binding motif (TAZ), β-catenin, and nuclear factor kappa B (NF-κB) [5]. Stiffening the extracellular matrix (ECM) induces integrin clustering and promotes focal adhesion kinase (FAK) phosphorylation. FAK triggers a sequence that activates RhoA and Rho kinase (ROCK), which then stimulate the assembly of contractile actin stress fibers. Studies have demonstrated that Rho activation through this pathway leads to increased YAP/TAZ activity [6,7].

YAP, as a converging effector of the Hippo pathway, is activated by dephosphorylation and is involved in cancer development [8]. TAZ is a paralog of YAP in vertebrates. When the Hippo pathway is turned off, YAP and TAZ are dephosphorylated and translocate to the nucleus. In the nucleus, YAP and TAZ combine with transcriptional enhanced associated domain (TEAD), the final nuclear effector of the Hippo pathway, and activate target genes, including CTGF, CYR61, AXL, and anti-apoptotic members of the Bcl2 and IAP families [9]. This eventually leads to the induction of cell growth, metabolism, proliferation, migration, and invasion [10,11]. YAP also plays a crucial role in the resistance of lung adenocarcinoma to epidermal growth factor receptor tyrosine kinase inhibitors (EGFR-TKIs) by increasing the expression of AXL [12]. In various types of cancer cells, it has been experimentally demonstrated that YAP regulates migration, cancer cell proliferation, control drug resistance, and cancer cell stemness in accordance with matrix stiffness [5,13,14,15,16,17]. Yuan et al. proved that matrix stiffness alters the behavior of non-small-cell lung cancer (NSCLC) cells and regulates the growth of NSCLC cells via YAP [17].

Programmed death ligand 1 (PD-L1, also known as B7-H1 or CD274) is an immune checkpoint protein that binds to its receptor PD-1 on T cells, B cells, and myeloid cells [18]. The PD-1/PD-L1 axis provides inhibitory signals that modulate the balance between T-cell activation, tolerance, and immunopathology [19,20]. The binding of PD-L1 to PD-1 on immune cells triggers an inhibitory response that facilitates immune evasion and progression [21]. In our previous study, we found that YAP plays an important role in the association between PD-L1 and EGFR-TKI resistance by directly regulating the expression level of PD-L1 in lung cancer in a PD1-independent manner [22]. A recent study revealed that PD-L1 can be regulated by mechanotransduction in lung cancer cells [23]. However, the specific mechanism of this intriguing regulation remains to be identified.

In this study, we aimed to determine whether YAP functions as a critical linker between matrix stiffness and PD-L1 in lung adenocarcinoma. In addition, we cultured lung cancer cells in a 3D environment, which is a more physiologically similar situation, and compared them with the results of 2D culture.

## 2. Materials and Methods

### 2.1. Transient Transfection of Cell Lines

The PC9 and PC9/GR human lung cancer cell lines were cultured in RPMI-1640 medium (WELGENE, Gyeongsan, Republic of Korea) supplemented with 10% fetal bovine serum (FBS) (WELGENE) at 37 °C in 5% CO_2_. The plasmids pDKflag-YAP wild-type (WT) and pDKflag-YAP2SA, a constitutively active YAP, and a control vector were provided by Prof. Dae Sik Lim of KAIST in Daejeon, South Korea. Lipofectamine 2000 (Invitrogen, Waltham, MA, USA) was used for the transfection of different DNA constructs according to the manufacturer’s instructions. After 48 h of incubation of transiently transfected cells, further assays were performed.

### 2.2. Polyacrylamide Hydrogel

First, 13 mm glass coverslips were activated by immersion in 0.1 M NaOH and allowed to dry. The coverslips were coated with an amine-reactive film using (3-aminopropyl) triethoxysilane 4.0% (Sigma-Aldrich, St. Louis, MO, USA) and then washed with distilled water for 10 min. After drying, the coverslips were coated with 2.5% glutaraldehyde (Sigma-Aldrich) for 30 min. They were then washed twice in distilled water for 10 min to produce a coating of aldehyde functional groups. Prepolymerization solutions containing varying ratios of acrylamide to bisacrylamide (Bio-Rad, Hercules, CA, USA) were utilized. The ratios used were 7.5:0.05 (1.6 kPa) and 12:0.24 (25.6 kPa) for %acrylamide–%bisacrylamide, respectively. The hydrogels were incubated in a sterile solution of dopamine hydrochloride at a concentration of 1 mg/mL in 50 mM HEPES buffer (pH 8.5) for 15 min to coat the gel surface with polymerized dopamine. The gels were washed three times with 50 mM HEPES (pH 8.5) to eliminate any remaining dopamine and then functionalized by incubating them for 30 min with 0.05 mg/mL sterile collagen I (PureCol, Advanced BioMatrix, Carlsbad, CA, USA) in Dulbecco’s phosphate-buffered saline (PBS).

For these experiments, commercially available polyacrylamide hydrogels of varying stiffness (0.5–32 kPa; Cytosoft 6-well plate, Advanced Biomatrix) were used. The hydrogels were bound to 6-well polystyrene plates or polystyrene dishes coated with type I collagen (C3867, Sigma-Aldrich).

### 2.3. Spheroid 3D Culture

PC9 and PC9/GR cells were 3D-cultured in Matrigel Basement Membrane Matrix (356237, Corning, Corning, NY, USA), and 10,000 cells of each cell type were dispersed in 20 μL of Matrigel. A drop of Matrigel was placed in the middle of the well of a 24-well plate, which was then inverted for the first 10 min of solidification, and 300 μL of culture medium was added and replaced every 3 days.

### 2.4. Western Blotting Assay

The cells were collected and suspended in a protein lysis buffer called Translab. The Bio-Rad Protein Assay (500-0006, Bio-Rad) was used to determine protein concentrations. A total of 30 µg of protein (µg/mL) was separated on a 10% SDS-PAGE gel. The protein was then transferred to a polyvinylidene difluoride (PVDF) membrane (Millipore, Burlington, MA, USA). The antibodies used in the study were anti-β-actin (sc-47778, Santa Cruz Biotechnology, Dallas, TX, USA), anti-YAP (#4912S, Cell Signaling, Danvers, MA, USA), and anti-PD-L1 (#13684, Cell Signaling). Blots were developed using an enhanced chemiluminescence detection kit (Bio-Rad).

### 2.5. siRNA-Mediated Gene Expression Knockdown

Small interfering RNA (siRNA) directed against YAP#1 (sense, 5′-CUG GUC AGA GAU ACU UCU UAA TT-3′; antisense, 5′-UUA AGA AGU AUC UCU GAC CAG TT-3′), YAP#2 (sense, 5′-GCC ACC AAG CUA GAU AAA GAT T-3′; antisense, 5′-UCU UUA YCU AGC UUG GUG GCT T-3′), p62#1 (sense, 5′-GAG GAU CCG AGU GUG AAU UUC CUC TT-3′; antisense, 5′-GAG GAA AUU CAC ACU CGG AUC CUC TT-3′), and negative control (sense, 5′-UUC UCC GAA CGU GUC ACG UTT-3′; antisense, 5′-ACG UGA CAC GUU CGG AGA ATT-3′) was synthesized by GenePharma. YAP siRNA (10 nM) was introduced into cells by transient transfection with RNAi MAX (Invitrogen) in accordance with the manufacturer’s instructions.

### 2.6. Immunofluorescence

Cultured cells were fixed with 4% paraformaldehyde at room temperature, permeabilized with 0.1% Triton X-100 in PBS, and blocked with 3% FBS in PBS. Immunofluorescence was detected using a fluorescence microscope (Leica) after overnight incubation at 4 °C with primary antibodies and incubation in the dark with Alexa 594 Fluor dye-labeled secondary antibodies.

### 2.7. RT-PCR

RNA was extracted from collected cells using TRIzol reagent (Invitrogen) according to the manufacturer’s instructions. Subsequently, cDNA was synthesized using Oli-go(dT) primers. The following primers were used for PCR amplification: (a) hYAP (sense, 5′-GAA CCA GAG AAT CAG TCA GA-3′; antisense, 5′-GGA TTG ATA TTC CGC ATT GC-3′), (b) hPD-L1 (sense, 5′-GGT GCC GAC TAC AAG CGA AT-3′; antisense, 5’-GGT GAC TGG ATC CAC AAC CAA-3’), (c) hCyR61 (sense, 5’-CCT TGT GGA CAG CCA GTG TA-3’; antisense, 5′-ACT TGG GCC GGT ATT TCT TC-3′), and (d) hβ-actin (sense, 5′-AGG CCC AGA GCA AGA GAG G-3′; antisense, 5′-TGC ATG GCT GGG GTG TTG AA-3′). The PCR products underwent electrophoresis on a 1% agarose gel and were visualized through ethidium bromide staining.

## 3. Results

### 3.1. Matrix Stiffness Regulates the Cell Proliferation and the Expiration of Both YAP and PD-L1 in Lung Adenocarcinoma

**Figure 1:**
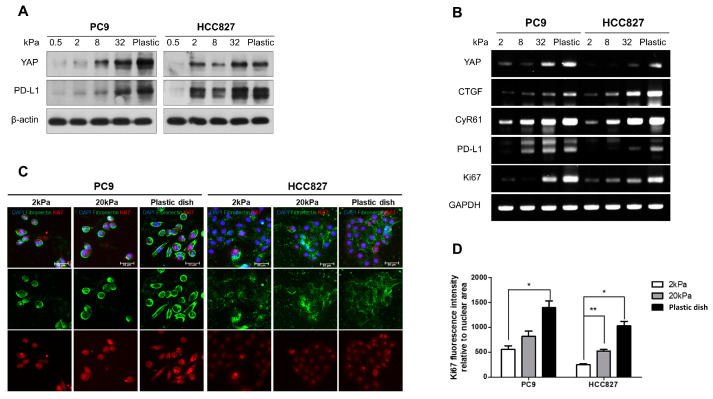
Regulation of substrate stiffness expression by YAP and PD-L1. (**A**) The effects of substrate stiffness on the expression of YAP and PD-L1 proteins in PC9 and HCC827 cells. Cells were cultured on polyacrylamide hydrogel substrates with varying rigidity, ranging from 0.5 to 32 kPa, and plastic dishes. (**B**) The effects of substrate stiffness on the expression of YAP, PD-L1, YAP downstream molecules (CTGF, CyR61), and Ki67 mRNA in PC9 and HCC827 cells. (**C**) Immunofluorescence demonstrated that fibronectin and Ki67 expression increased with the rise in matrix stiffness. Scale bars indicate 50 μm. (**D**) The Ki67 fluorescence intensity related to the nuclear area was quantified and expressed as a bar graph. Student’s *t*-test (two-tailed *p*-value) was used, and *p*-values * *p* < 0.05, ** *p* < 0.01 were considered significant. The uncropped blots are shown in Supplementary Materials.

To examine the impact of matrix stiffness on lung adenocarcinoma cell lines, PC9 and HCC827, we cultured the cells in collagen-coated polyacrylamide hydrogel with stiffnesses ranging from 0.5 kPa to plastic (approximately 1 GPa). The results indicated that the expression levels of the YAP and PD-L1 proteins increased as the matrix stiffness increased in both cell lines (Figure 1A). To determine whether matrix stiffness regulates cell proliferation and the expression of YAP and PD-L1, we measured the mRNA levels of YAP, PD-L1, Ki-67 (as a proliferation marker), and YAP downstream molecules, such as CTGF and CyR61, using real-time PCR (RT-PCR). Increased expression of YAP, PD-L1, YAP downstream molecules, and Ki-67 was observed in a stiffer matrix (Figure 1B). In addition, we performed immunofluorescence staining to confirm the expression of Ki-67 and fibronectin, a fibrotic marker, in gels with different degrees of matrix stiffness. We found that fibronectin and Ki-67 were upregulated in accordance with the increase in matrix stiffness (Figure 1C,D). The data indicate that matrix stiffness regulates cell proliferation and the expression of YAP and PD-L1.

### 3.2. YAP Plays an Important Role in Regulating the PD-L1 Expression and Proliferating Capacity in the Stiff Matrix

**Figure 2:**
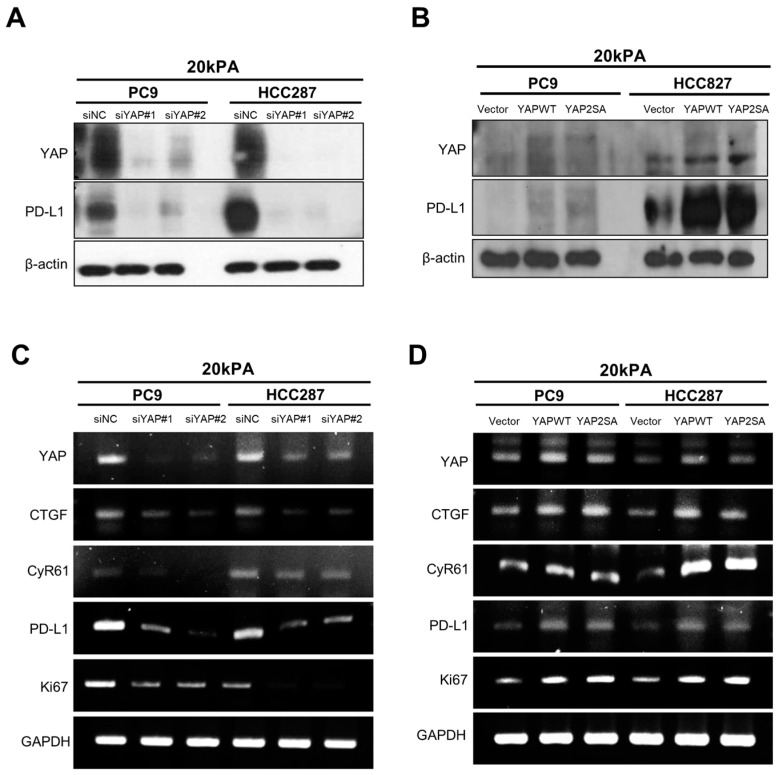
YAP regulates the expression of PD-L1 in lung cancer in a matrix with high stiffness. (**A**) Knockdown of YAP in 20 kPa matrix was confirmed by Western blot analysis. (**B**) Overexpression of YAP in 20 kPa matrix was confirmed by Western blot analysis. (**C**) Inhibition of YAP, PD-L1, YAP downstream molecules (CTGF, CyR61), and Ki67 expression level in 20 kPa matrix was confirmed by RT-PCR analysis. (**D**) Overexpression of YAP, PD-L1, YAP downstream molecules (CTGF, CyR61), and Ki67 mRNA in 20 kPa matrix was confirmed by RT-PCR analysis. The uncropped blots are shown in Supplementary Materials.

To confirm the effect of YAP knockdown on the increased expression of YAP and PD-L1 in the stiff matrix (20 kPa), cells transfected with siYAP were compared. The results showed that the knockdown of YAP dramatically reduced the expression level of PD-L1 in the stiff matrix (Figure 2A). Similarly, the mRNA expression of downstream molecules of YAP and PD-L1, as well as Ki-67, was significantly decreased in YAP-depleted cells (Figure 2B). Conversely, when YAP2SA, an active YAP mutant that overexpresses YAP, was cultured in a stiff matrix, the protein production of PD-L1 was increased (Figure 2C), and the mRNA expression of PD-L1 and Ki-67 was also increased (Figure 2D). These results indicate that PD-L1 expression and cell proliferation can be controlled by YAP in a stiff matrix.

### 3.3. YAP Regulates the Expression of PD-L1 and Cell Proliferation in 3D Culture Conditions

**Figure 3:**
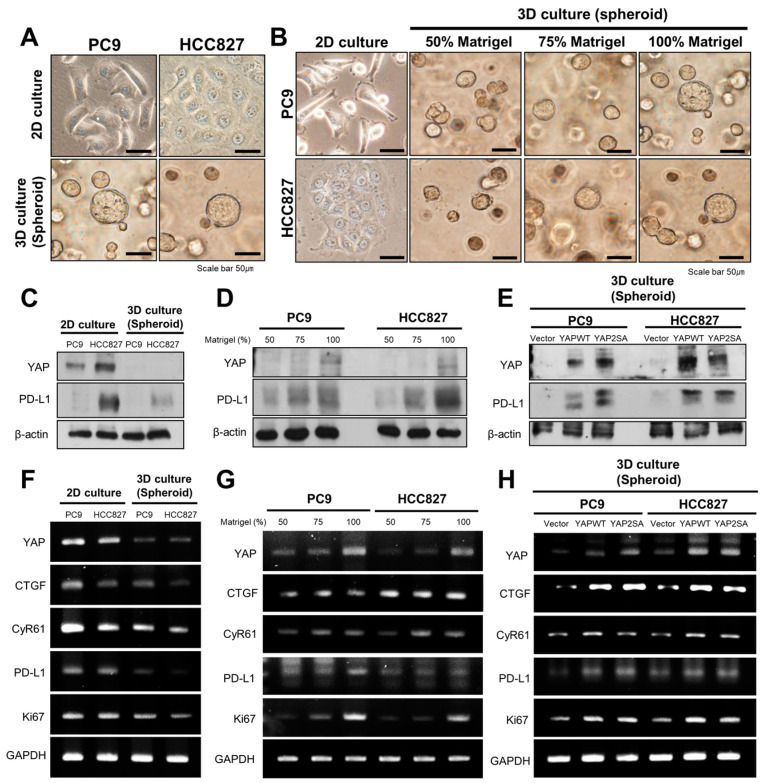
Expression of YAP and PD-L1 and the cell growth of 2D- and 3D-cultured lung cancer cell lines. (**A**) Bright-field images of PC9 and HCC827 cells grown in 2D and 3D conditions were captured 5 days after cell seeding. Scale bars indicate 50 μm. (**B**) Bright-field image according to Matrigel concentration of PC9 and HCC827 cells. (**C**) Western blot analysis for expression of YAP and PD-L1 proteins in 2D and 3D conditions. (**D**) Western blot analysis for expression of YAP and PD-L1 proteins according to Matrigel concentration. (**E**) Overexpression of YAP in 3D conditions was confirmed by Western blot analysis. (**F**) RT-PCR analysis for expression of YAP, PD-L1, and CyR61 mRNAs. (**G**) RT-PCR analysis for expression of YAP, PD-L1, YAP downstream molecules (CTGF, CyR61), and Ki67 according to Matrigel concentration. (**H**) Overexpression of YAP, PD-L1, YAP downstream molecules (CTGF, CyR61), and Ki67 mRNA in 3D culture was confirmed by RT-PCR analysis. The uncropped blots are shown in Supplementary Materials.

We also used 3D culture, which is a more physiologically similar environment, to check whether there would be a difference from the result obtained by 2D culture. When PC9 and HCC 827 cells were cultured for 5 days in 2D and 3D conditions, they showed a stretched shape in 2D culture but showed a spherical shape in 3D culture with 100% Matrigel (Figure 3A). We continued the 3D culture by adjusting the concentration of Matrigel from 50% to 100% to investigate whether there were morphological changes in the cells according to the concentration of Matrigel. We found that cell spheroids grew larger with increasing Matrigel concentration (Figure 3B). We compared the expression levels of YAP and PD-L1 in 2D and 3D cultures. We observed that the protein expression of YAP and PD-L1 was reduced in 3D culture compared to 2D culture using Western blotting (Figure 3C). Consistent with this, the mRNA expression of YAP, PD-L1, and Ki-67 was lower in 3D culture than in 2D culture (Figure 3F).

To investigate the change in the expression of YAP and PD-L1 depending on the concentration of Matrigel, PC9 and HCC827 cells were cultured with Matrigel at different concentrations from 50% to 100%. As the concentration of Matrigel increased, the protein expression of YAP and PD-L1 increased (Figure 3D), and the mRNA expression of YAP, PD-L1, YAP downstream molecules (CTGF, CyR61), and Ki-67 was also upregulated (Figure 3G). The YAP2SA mutant was used to determine the level of protein and mRNA expression of YAP and PD-L1 when YAP was overexpressed in 3D culture. In 3D conditions, consistent with the 2D condition culture, the expression levels of YAP, PD-L1, and Ki-67 were markedly increased when YAP was overexpressed (Figure 3E,H). Similar to the results of 2D culture, it was confirmed that YAP, PD-L1, and Ki-67 were upregulated with increasing substrate stiffness.

## 4. Discussion

Matrix stiffness has been identified as an important factor regulating tumor progression and metastasis [24,25,26,27,28]. An increase in tissue stiffness is induced by ROCK activation and actomyosin-mediated cellular contractility, leading to β-catenin-mediated hyperproliferation and increased tumor burden and progression [29]. Matrix stiffening induces the epithelial–mesenchymal transition (EMT) through the activation of the TGF-β signaling pathway and results in a more aggressive tumor phenotype [20,30]. Matrix stiffness turns fibroblasts into cancer-associated fibroblasts and plays a role in maintaining this state through YAP, a mechanosensitive transcription factor that also affects the stromal cells around tumor cells [8].

YAP and TAZ are transcriptional coactivators that shuttle between the cytoplasm and the nucleus. Their activation has been linked to the induction of cancer stem cell properties, proliferation, chemoresistance, and metastasis [31,32,33,34,35,36,37]. The analysis of a cohort of NSCLC patients showed that those with higher tumor TAZ expression had poorer prognoses [38]. YAP is a sensor of the structural and mechanical features of the cell microenvironment. The role of YAP in mechanotransduction is attributed to its capacity to directly promote the transcription of genes that are involved in cell–matrix interactions, ECM composition, and cytoskeleton integrity [39]. A stiff matrix and contractile actin cytoskeleton work together to activate YAP. In addition, YAP is required for the stiffening of the matrix by cancer-associated fibroblasts, thereby creating a positive feedback loop [8]. According to Yuan and colleagues, YAP may act as an intermediary between the growth of NSCLCs and their environment [17]. Furthermore, Miyazawa et al. also established that PD-L1 expression and cell growth were affected by matrix stiffness in human lung cancer cells [23].

**Figure 4:**
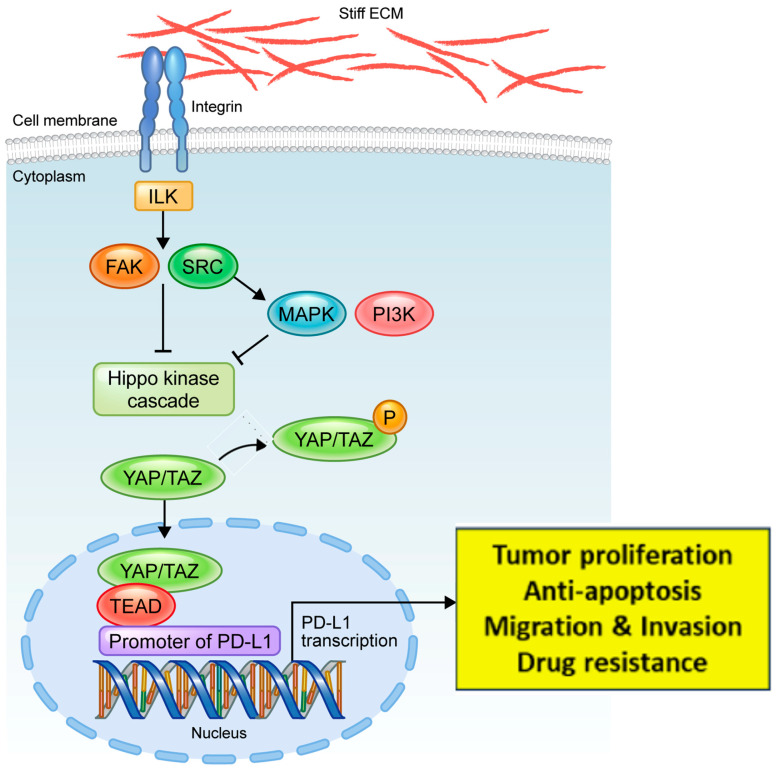
Schematic illustration of the effect of matrix stiffness on YAP and PD-L1 expression and tumor cell behavior.

Our previous study showed that YAP directly regulates the expression of PD-L1 transcripts and that the YAP/TEAD complex binds to the PD-L1 promoter in EGFR-TKI-resistant lung adenocarcinoma [22]. Therefore, we investigated whether YAP regulates the expression of PD-L1 in proportion to the matrix stiffness. We demonstrated that, as the matrix stiffness increased, the expression levels of YAP and PD-L1 increased proportionally. We also found that the knockdown of YAP significantly reduced the expression of PD-L1 under stiff-matrix conditions (20.0 kPa). In the same context, it was confirmed that the overexpression of YAP by the YAP2SA mutant led to an increase in the expression of PD-L1 and Ki-67. Similar results were also obtained with 3D culture using Matrigel. Based on our data, we conclude that the stiffness of the matrix regulates the expression of PD-L1 via YAP activation and is ultimately involved in the proliferation of cells (Figure 4).

To compare 2D culture results, we grew PC9 and HCC827 cells in 3D cultures, which are more physiologically similar to in vivo conditions. In both 2D and 3D environments, an increase in the expression of YAP and PD-L1 was observed as the matrix became stiffer. This is confirmed in Figure 1 for the 2D environment and in Figure 3 for the 3D spheroid culture. The stiffness in 3D cultures was adjusted by varying the concentration of Matrigel. In 2D culture, the cells attached and spread along the base, but when they were cultured in 3D conditions, they formed spherical shapes. This result suggests that the mechanical stress applied to the cells differed between the two culture methods. The cultivation of cells in 3D conditions resulted in decreased protein and mRNA expression levels of YAP and PD-L1 compared to those in 2D culture (Figure 3C,F). A bioengineering technique was employed to create a spatial model of YAP/TAZ, which confirmed that as the nuclear shape becomes flatter, the nuclear/cytoplasmic ratio of YAP increases [6]. Our study confirmed that cells cultivated in 2D displayed a flattened morphology. In 2D culture, the flattening of the nucleus promoted YAP expression. However, in 3D culture, the cells lacked adherence to a base, resulting in less YAP expression than in 2D culture.

This study confirms that an increase in matrix stiffness leads to the upregulated expression of YAP and PD-L1. Since the upregulation of PD-L1 can promote tumor proliferation, anti-apoptosis, drug resistance, and invasion, compounds that modulate matrix stiffness or target upstream molecules such as YAP might have potential as drugs for the treatment of lung cancer. In previous studies, it was observed that treatment with the YAP inhibitor verteporfin resulted in a decrease in cell viability [12,40]. Additionally, a decrease in both YAP and PD-L1 expression was confirmed. It is possible that the use of YES inhibitors and YAP inhibitors, either alone or in conjunction with current anticancer medications, could improve the anticancer effects.

## 5. Conclusions

In summary, this study demonstrates the significant role of YAP in linking the TME and PD-L1. These findings indicate that the proliferation and invasiveness of lung cancer cells, which are mediated by YAP and PD-L1, are affected by the matrix stiffness, a crucial component of the TME.

## Data Availability

The data presented in this study are contained within the article.

## Supplementary Materials

The following supporting information can be downloaded at https://www.mdpi.com/article/10.3390/cancers16030598/s1. Western blot information.
