# Supplementary material for: The Matrix Stiffness Coordinates the Cell Proliferation and PD-L1 Expression via YAP in Lung Adenocarcinoma
Source: Cancers (Basel). 2024 Jan 31;16(3):598. doi: 10.3390/cancers16030598 (PMC10854616; doi:10.3390/cancers16030598)
Supplement: Supplementary file 1 [file cancers-16-00598-s001.zip › cancers-2788056-supplementary.pdf]

Figure 1A

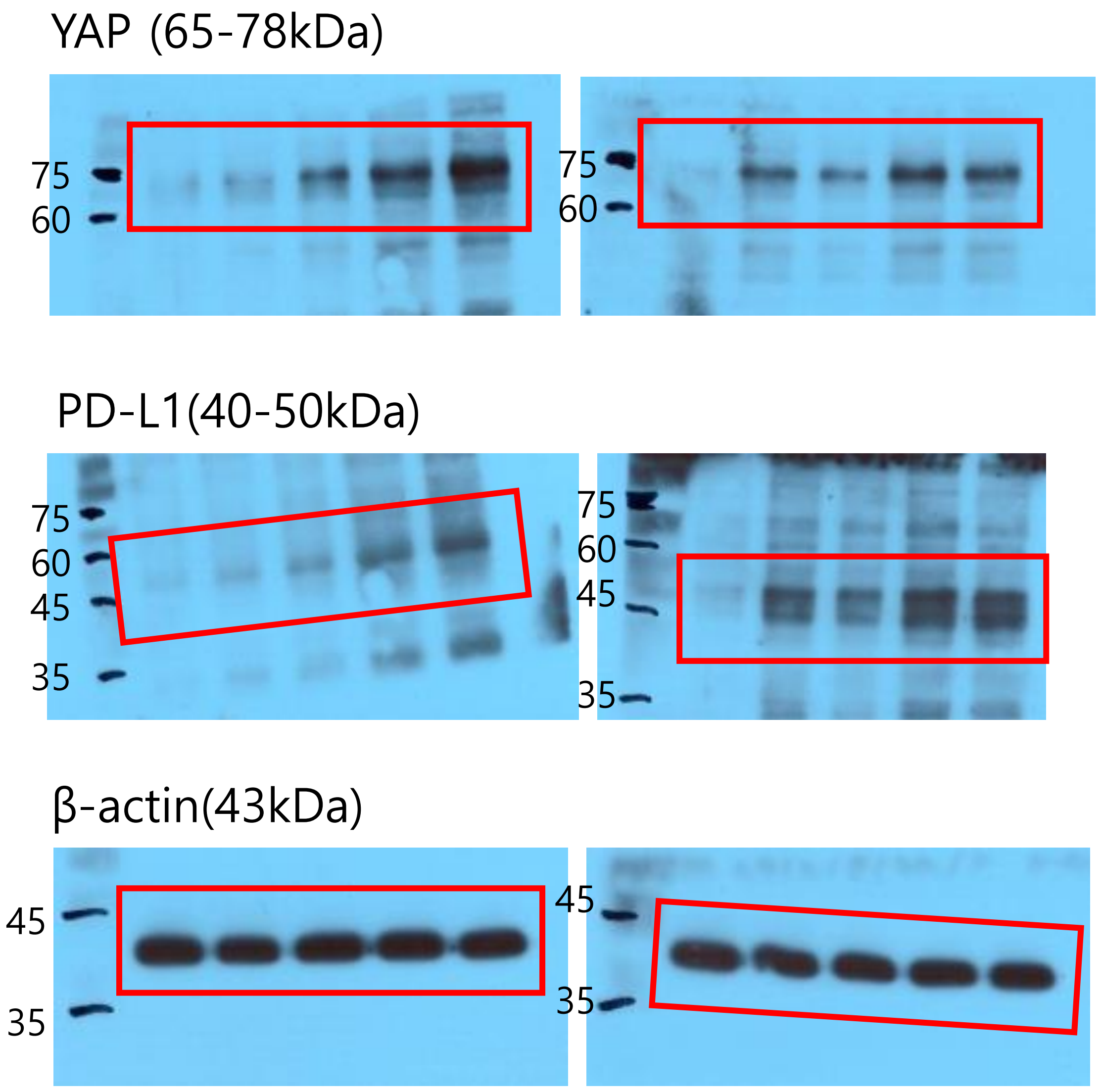
YAP (65-78kDa)
75
60
75
60
PD-L1(40-50kDa)
75
60
45
35
75
60
45
35
β-actin(43kDa)
45
35
45
35

Figure 1B

hKi67 sense, 5′-ACG AGA CGC CTG GTT ACT ATC-3′; antisense, 5′-GCT CAT CAA TAA CAG ACC CAT TTA C-3′
hCTGF sense, 5′-ACC GAC TGG AAG ACA CGT TTG-3′; antisense, 5′-CCA GGT CAG CTT CGC AAG G-3′

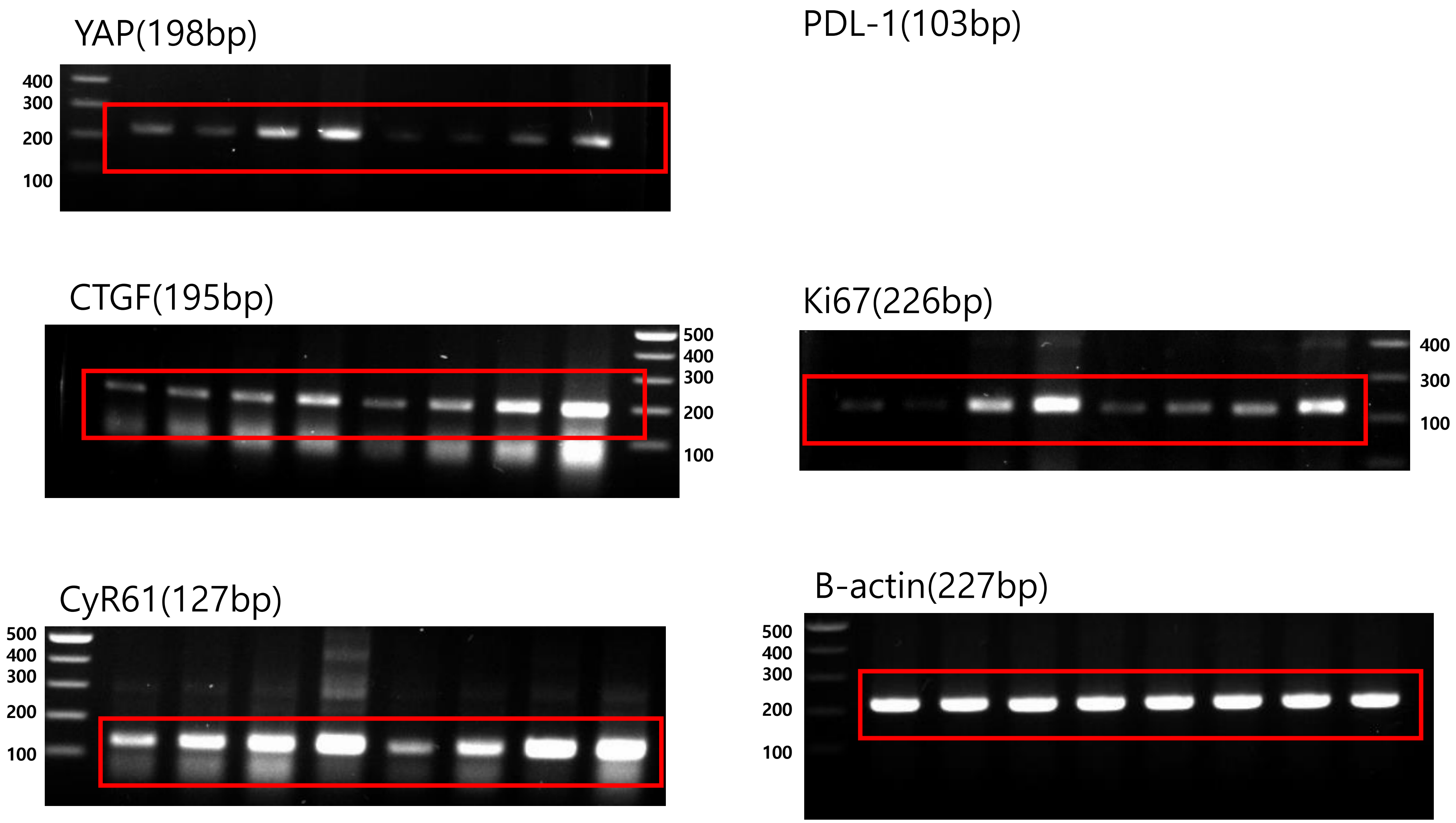

Figure 2A

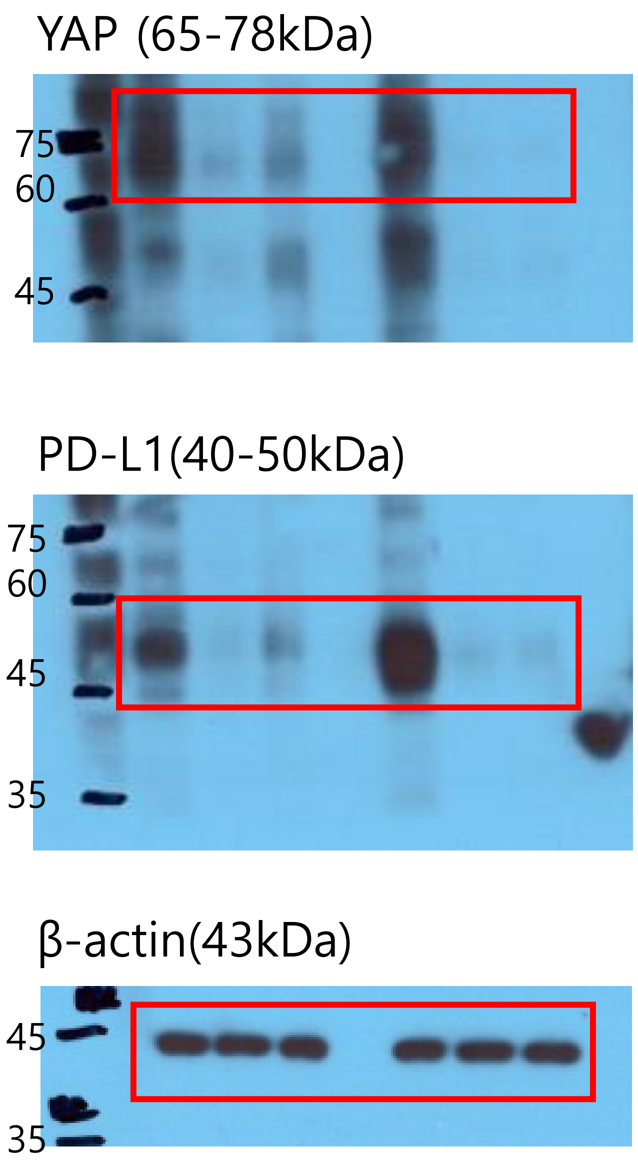

Figure 2B

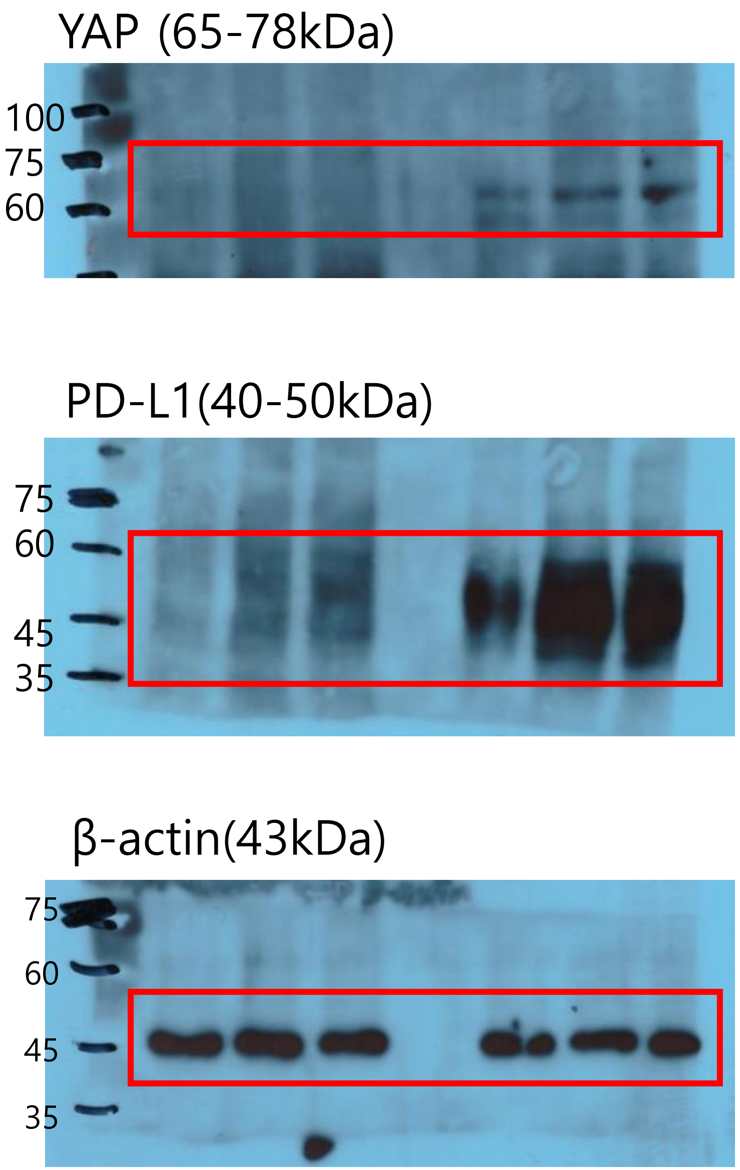

Figure 2C

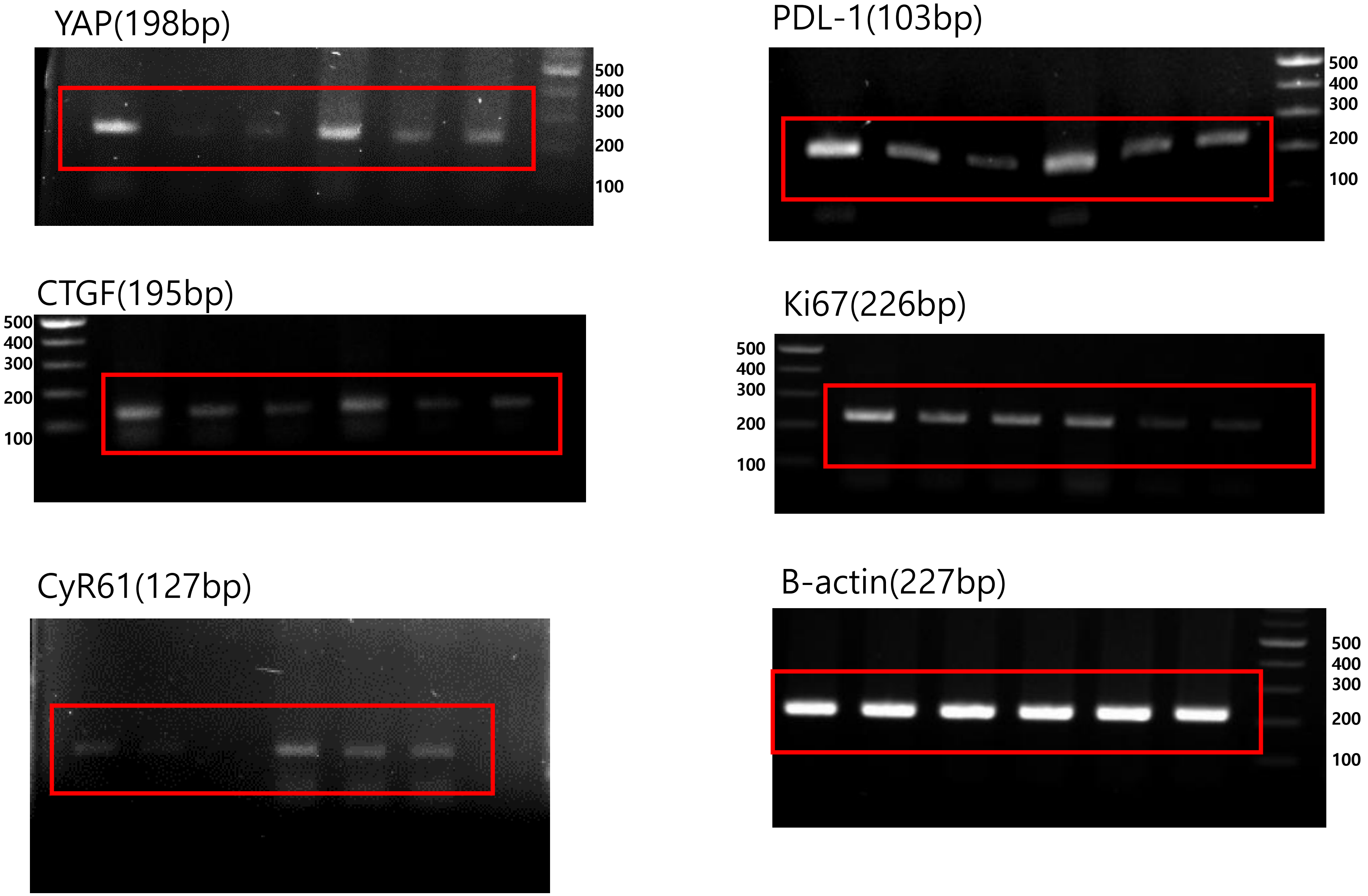

YAP(198bp)
500
400
300
200
100
PDL-1(103bp)
500
400
300
200
100
CTGF(195bp)
500
400
300
200
100
Ki67(226bp)
500
400
300
200
100
CyR61(127bp)
B-actin(227bp)
500
400
300
200
100

Figure 2D

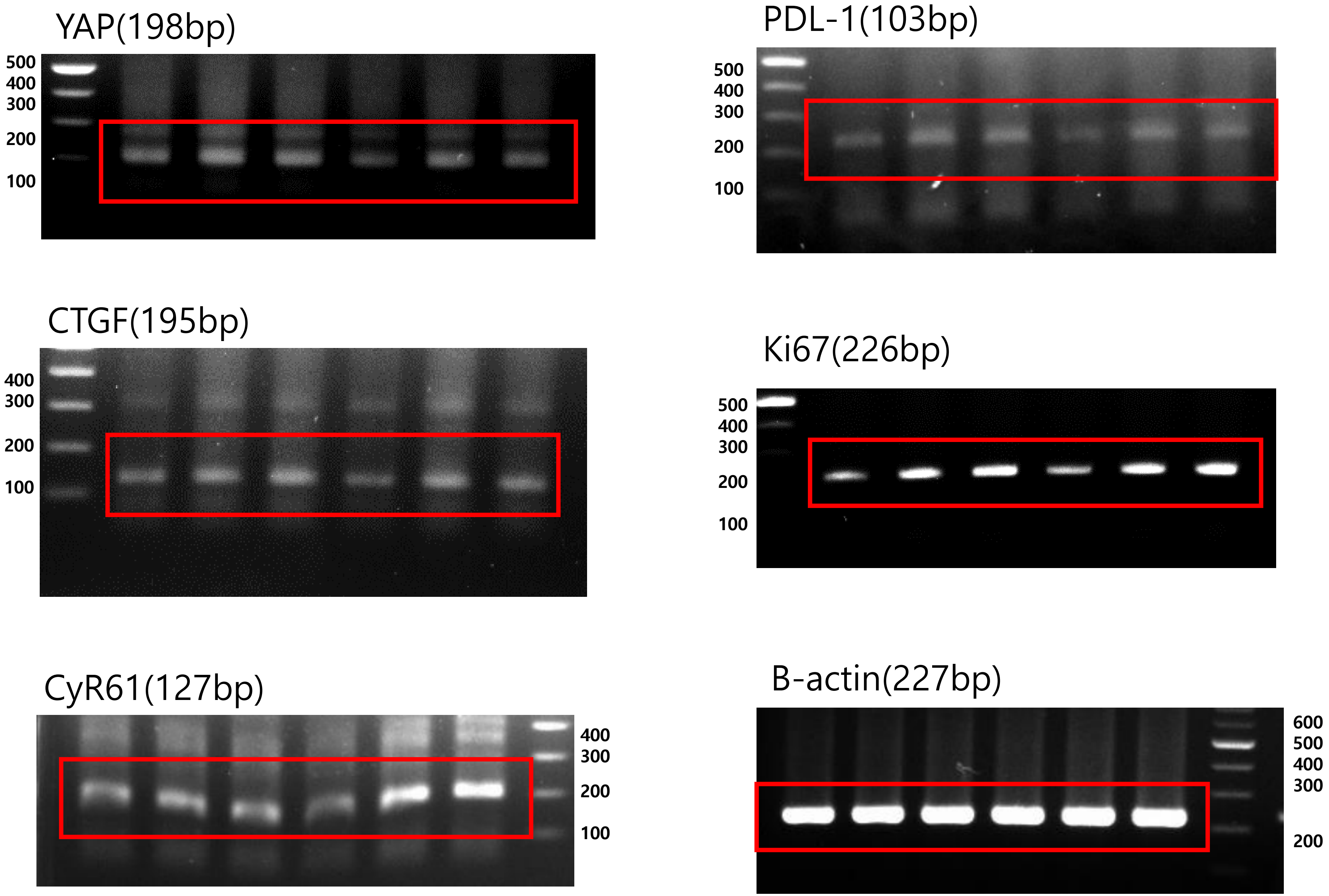
YAP(198bp)
500
400
300
200
100
PDL-1(103bp)
500
400
300
200
100
CTGF(195bp)
400
300
200
100
Ki67(226bp)
500
400
300
200
100
CyR61(127bp)
400
300
200
100
B-actin(227bp)
600
500
400
300
200

Figure 3C

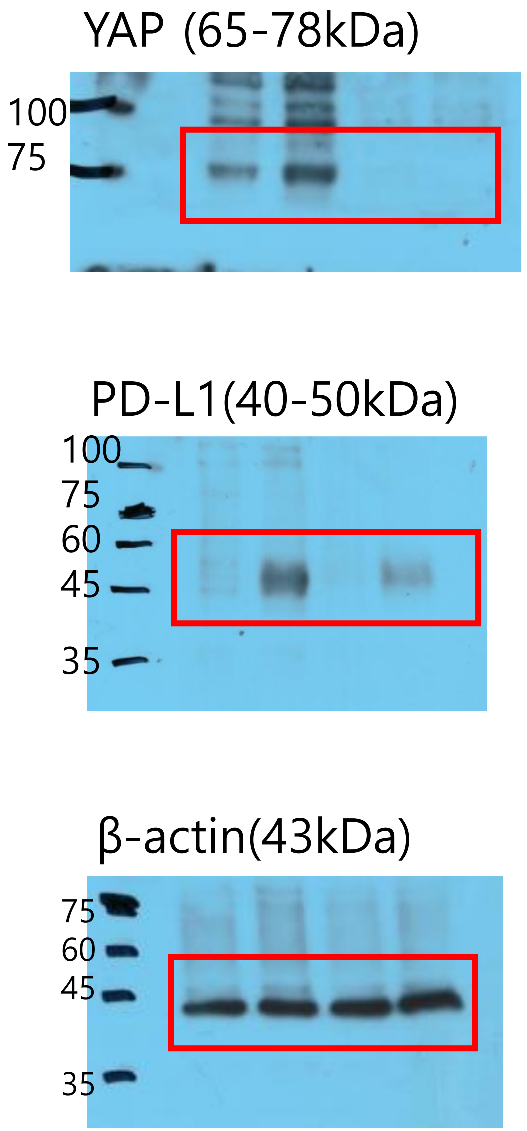

Figure 3D

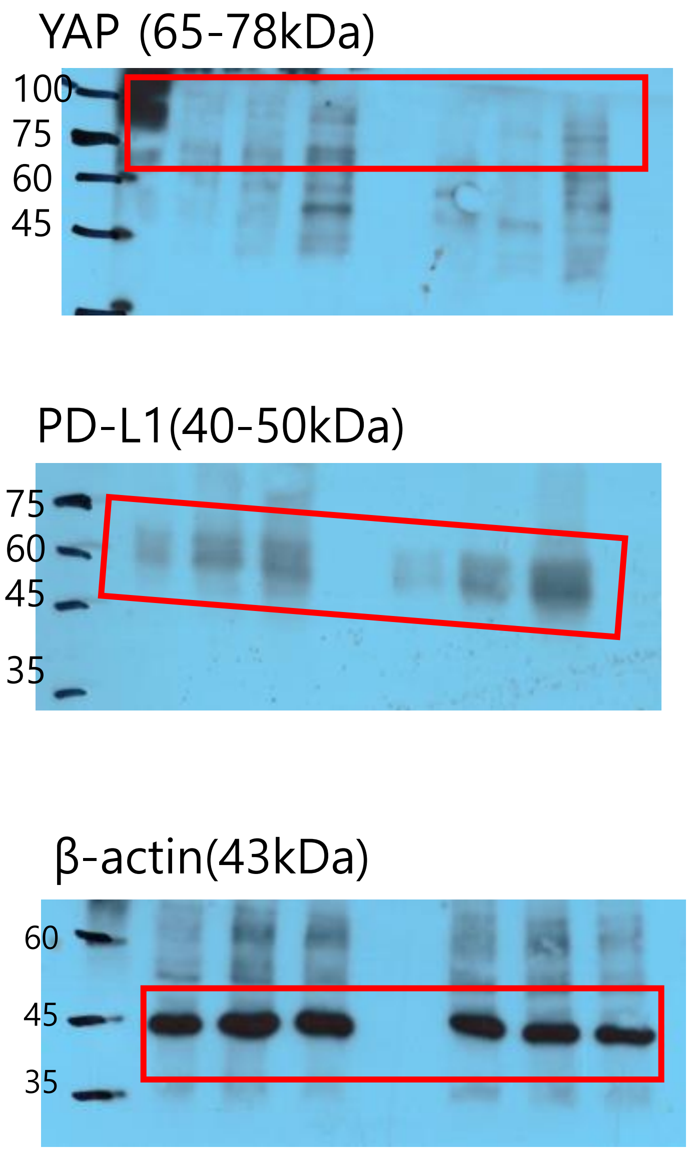

Figure 3E

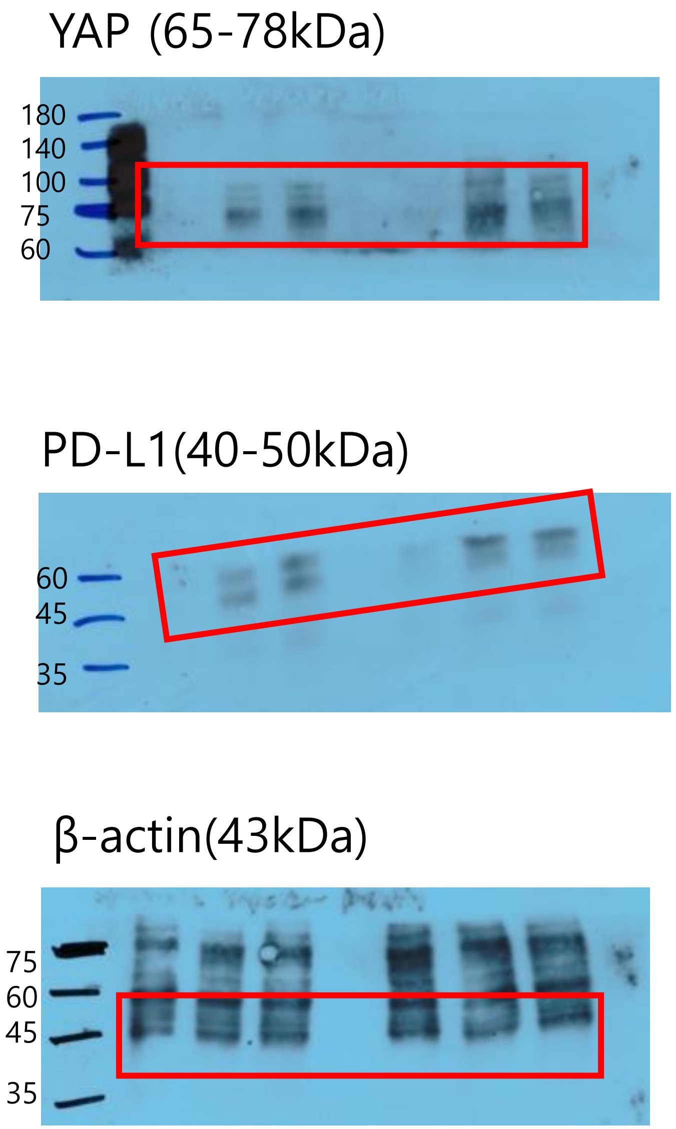

Figure 3F

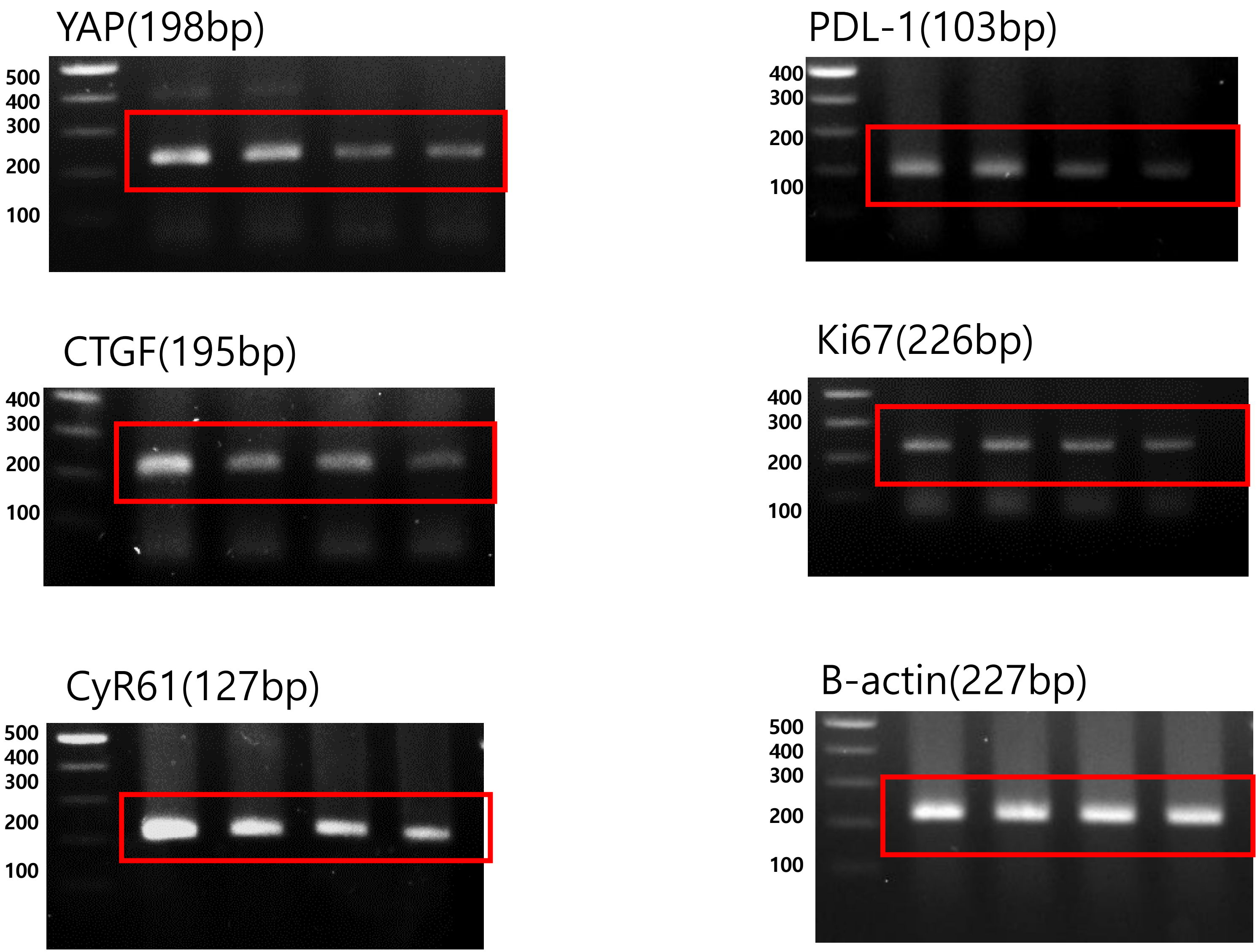
YAP(198bp)
500
400
300
200
100
PDL-1(103bp)
400
300
200
100
CTGF(195bp)
400
300
200
100
Ki67(226bp)
400
300
200
100
CyR61(127bp)
500
400
300
200
100
B-actin(227bp)
500
400
300
200
100

Figure 3G

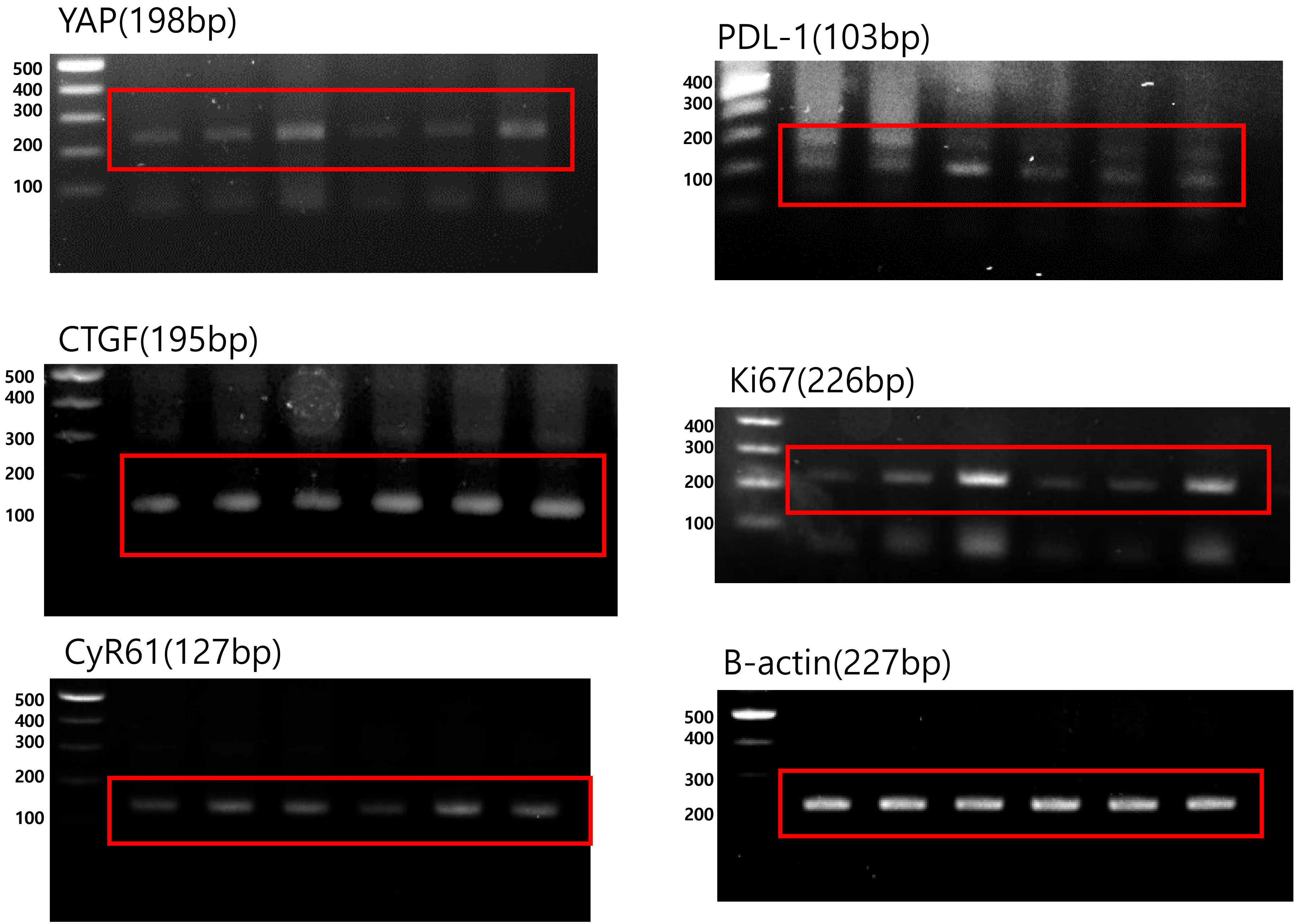

YAP(198bp)
500
400
300
200
100
PDL-1(103bp)
400
300
200
100
CTGF(195bp)
500
400
300
200
100
Ki67(226bp)
400
300
200
100
CyR61(127bp)
500
400
300
200
100
B-actin(227bp)
500
400
300
200

Figure 3H

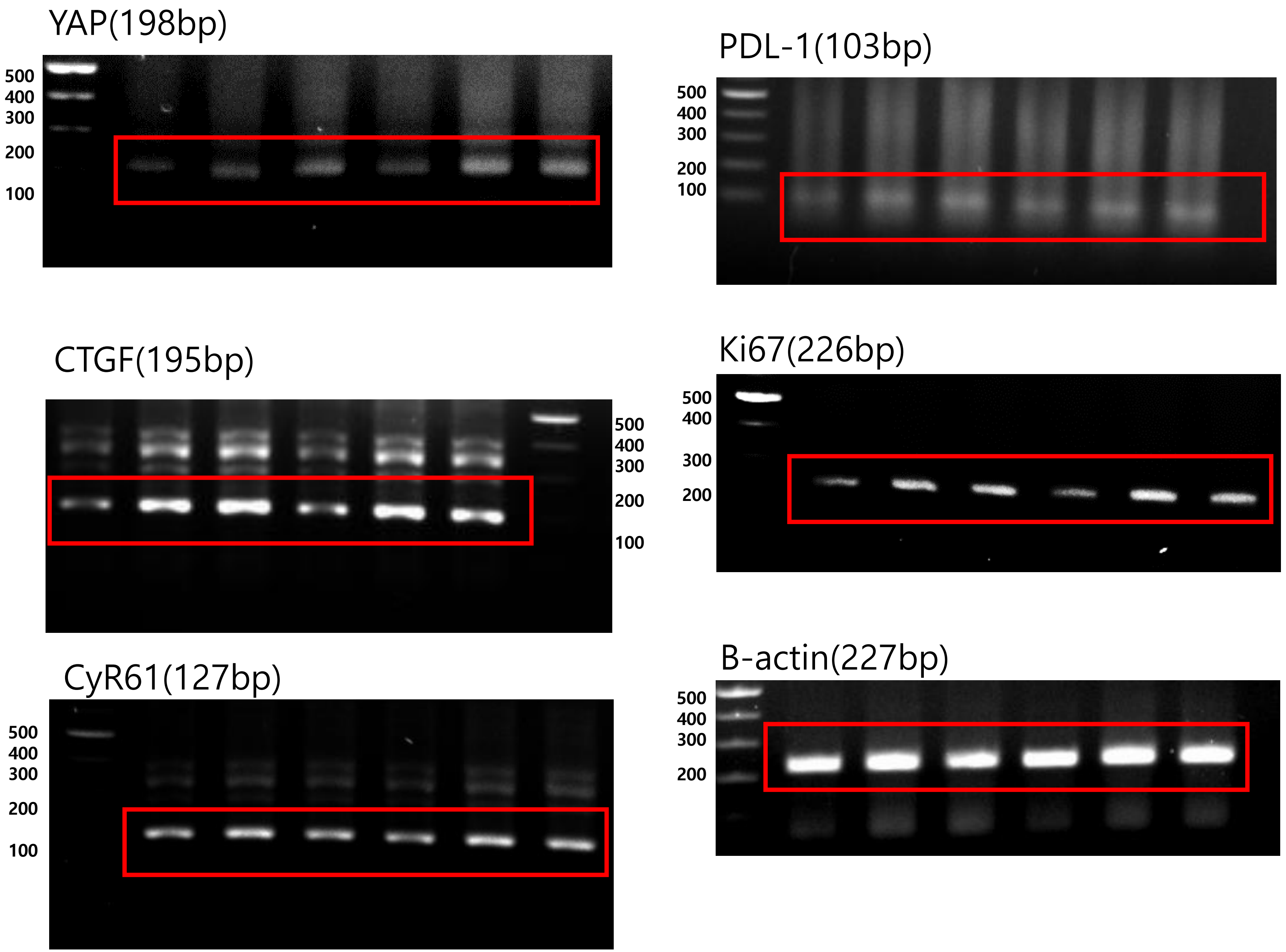

YAP(198bp)
500
400
300
200
100
PDL-1(103bp)
500
400
300
200
100
CTGF(195bp)
500
400
300
200
100
Ki67(226bp)
500
400
300
200
CyR61(127bp)
500
400
300
200
100
B-actin(227bp)
500
400
300
200
